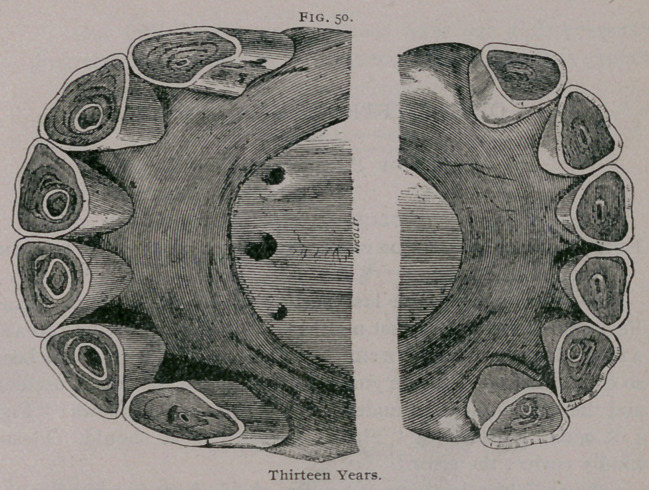# Age of the Horse, Ox, Dog, and Other Domesticated Animals

**Published:** 1890-12

**Authors:** R. S. Huidekoper

**Affiliations:** Veterinarian


### AGE OF THE HORSE, OX, DOG, AND OTHER DOMESTICATED ANIMALS.

BY R. S. HUIDEKOPER, M.D., VETERINARIAN.

[*Continued from page* 612.]

FIG. 47.

FIG. 47.

Ten Years.

*Ten Years.*—FIG. 47.

FROM the increased obliquity of the teeth the jaws become prominent in front, and at the ordinary height of the head the inferior incisors are hidden when looked at from in front. The ogive formed by the two sets of incisors when looked at in profile is more closed. The angle of the teeth increases, and a greater inter-space is found between the intermediate and corner teeth. On the tables the inferior pinchers are seen to be more rounded than at nine years, the cup is smaller and decidedly triangular. It is closer to the posterior border of the teeth ; the dental star, still more apparent, is found about the centre of the teeth. The intermediate teeth are rounded and the corner teeth commence to assume this form. The example furnished by the plate shows deep fissures on the posterior border of the corner teeth. The incisive arch is flatter in the centre. In the plate showing the teeth from in front the superior pinchers are seen to be somewhat worn from cribbing.

Fig. 48.

FIG. 48.

Eleven Years.

*Eleven Years.*—FIG. 48.

The incidence of the jaws increases in obliquity, so that the head of the horse must be raised to see both from in front. In profile, the superior corner tooth is decidedly more oblique than the intermediate one, the inferior is the same size at its free surface and its base, and its gum is square. The inferior tables are round on the intermediate and rounded on the corners. In the lower jaw the cups are diminished to little islands close to the posterior borders of the tables, and the dental stars are narrower from side to side and near the centre of the tables. In the upper jaw the cups of the corner teeth are elliptic and commence to disappear.

Fig. 49.

FIG 49.

Twelve Years.

*Twelve Years.*—FIG. 49.

The incidence of the jaws is still more oblique than at eleven years. In profile, the superior corner tooth is still more oblique. It has a notch on its posterior border, and it is separated further from the intermediate. The inferior tables are round and nearly leveled, or only have traces of the cups. A yellow spot represents the dental star. The superior corners are almost leveled. The incisive arches are narrower and less convex than at an earlier age.

FIG. 50.

FIG. 50.

Thirteen Years.

In front the appearance is much the same as it is at twelve years. In profile, the notch of the superior corner is larger, the inferior corner appears narrow, with its borders parallel. The plate, drawn from a mare's mouth, shows rudimentary tusk teeth. The inferior tables are round and generally leveled. The superior corners are generally leveled. In the superior pincers the cups are rounded. [TO BE CONTINUED.]

## Figures and Tables

**Fig. 47. f1:**
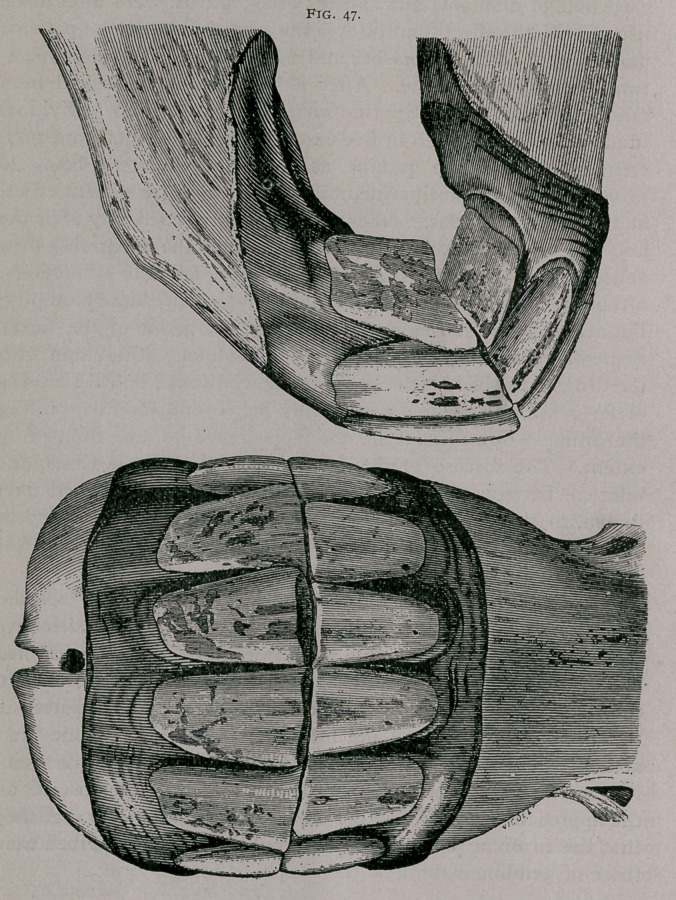


**Fig. 47. f2:**
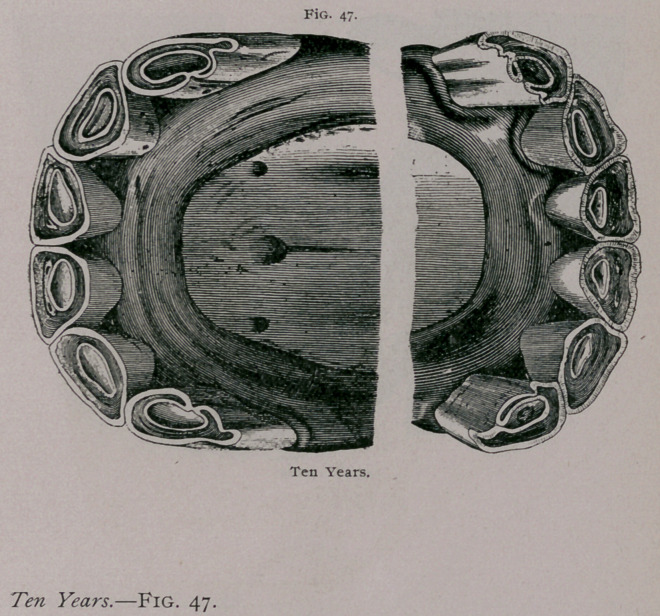


**Fig. 48. f3:**
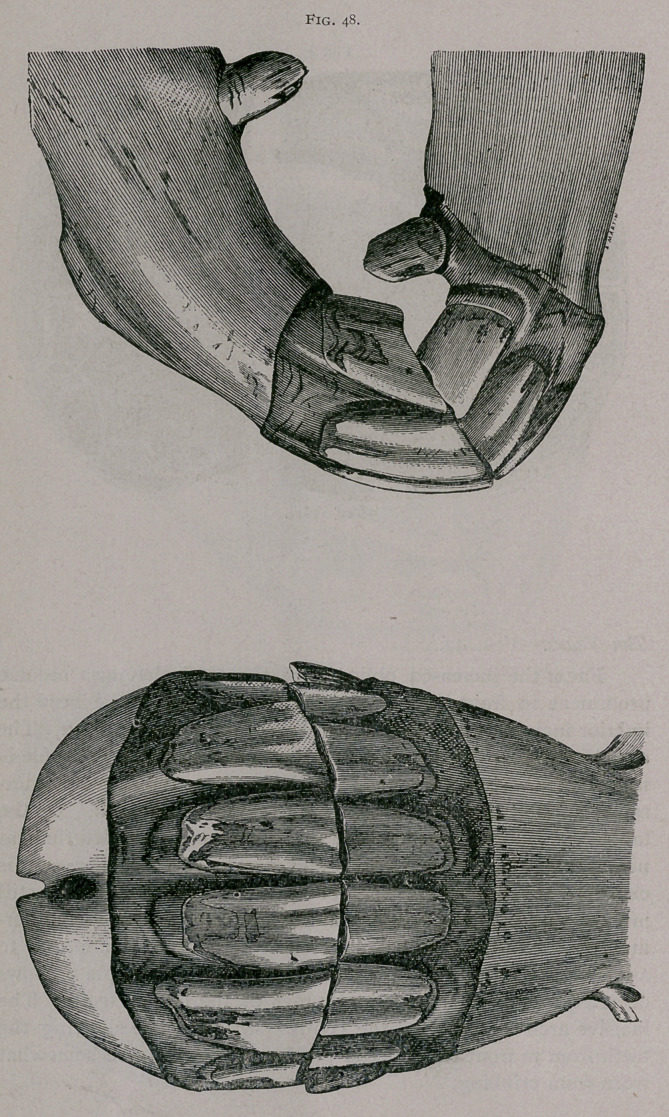


**Fig. 48. f4:**
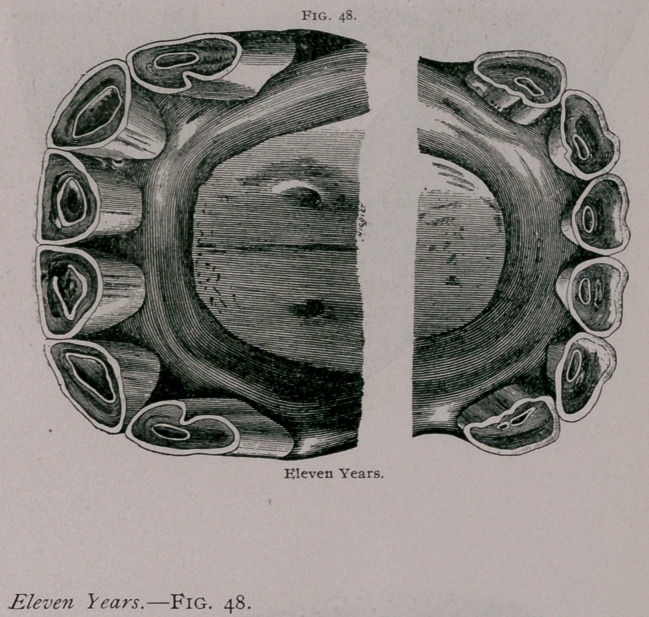


**Fig. 49. f5:**
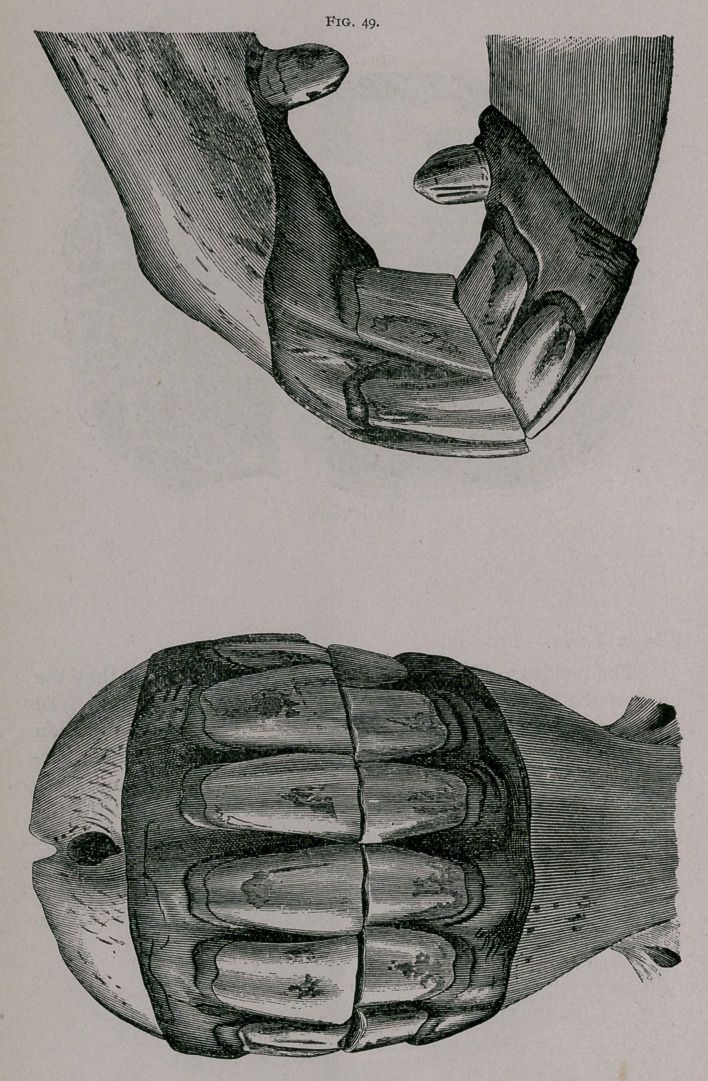


**Fig. 49. f6:**
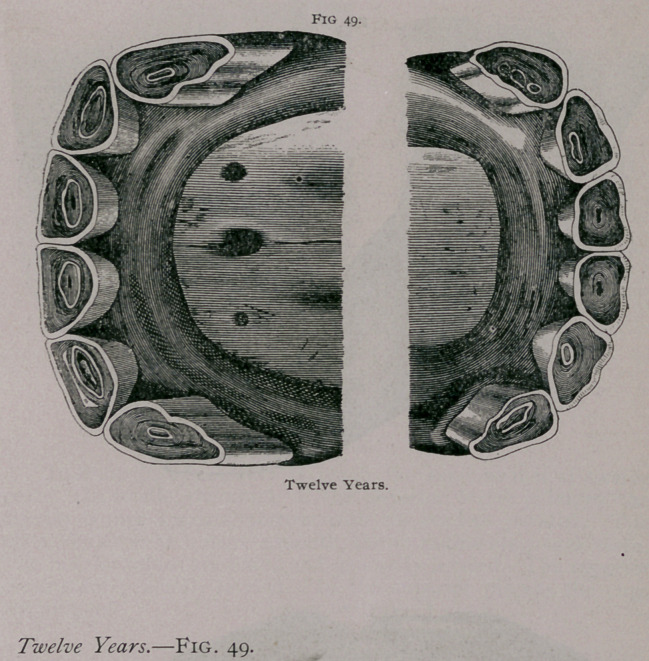


**Fig. 50. f7:**
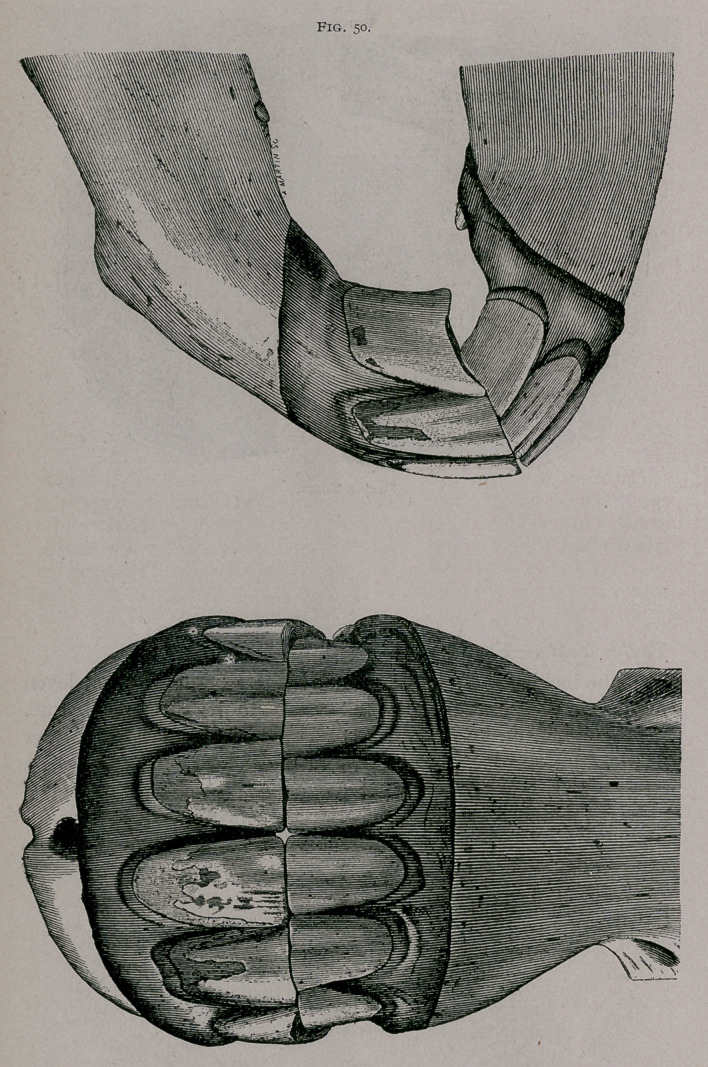


**Fig. 50. f8:**